# Serum and Bronchoalveolar Lavage Fluid Levels of Cytokines in Patients with Lung Cancer and Chronic Lung Disease: A Prospective Comparative Study

**DOI:** 10.3390/jpm13060998

**Published:** 2023-06-14

**Authors:** Patricia Hogea, Emanuela Tudorache, Ovidiu Fira-Mladinescu, Monica Marc, Diana Velescu, Diana Manolescu, Felix Bratosin, Ovidiu Rosca, Adelina Mavrea, Cristian Oancea

**Affiliations:** 1 Center for Research and Innovation in Personalized Medicine of Respiratory Diseases, “Victor Babes” University of Medicine and Pharmacy, Eftimie Murgu Square 2, 300041 Timisoara, Romania; hogea.patricia@umft.ro (P.H.); tudorache_emanuela@yahoo.com (E.T.); mladinescu@umft.ro (O.F.-M.); marc.monica@umft.ro (M.M.); velescu.diana@umft.ro (D.V.); dmanolescu@umft.ro (D.M.); oancea@umft.ro (C.O.); 2 Doctoral School, “Victor Babes” University of Medicine and Pharmacy, Eftimie Murgu Square 2, 300041 Timisoara, Romania; 3 1st Pulmonology Clinic, Clinical Hospital of Infectious Diseases and Pulmonology, “Victor Babes”, Gheorghe Adam Street 13, 300310 Timisoara, Romania; 4 2nd Pulmonology Clinic, Clinical Hospital of Infectious Diseases and Pulmonology, “Victor Babes”, Gheorghe Adam Street 13, 300310 Timisoara, Romania; 5 Discipline of Radiology, “Victor Babes” University of Medicine and Pharmacy Timisoara, Eftimie Murgu Square 2, 300041 Timisoara, Romania; 6 Discipline of Infectious Diseases, “Victor Babes” University of Medicine and Pharmacy Timisoara, Eftimie Murgu Square 2, 300041 Timisoara, Romania; felix.bratosin@umft.ro (F.B.); ovidiu.rosca@umft.ro (O.R.); 7 Department of Internal Medicine I, Cardiology Clinic, “Victor Babes” University of Medicine and Pharmacy Timisoara, Eftimie Murgu Square 2, 300041 Timisoara, Romania

**Keywords:** lung cancer, interleukins, cytokines, bronchial lavage

## Abstract

The role of chronic inflammation in the initiation and progression of carcinogenesis has been well-established in previous studies, particularly in the stages of malignant conversion, invasion, and metastasis. This study aimed to explore the potential correlation between the levels of cytokines in serum and bronchoalveolar lavage fluid (BALF) by comparing their levels between patients with lung cancer and those with benign lung diseases. The study measured the concentration of IFN-γ, TNF-α, IL-1β, IL-2, IL-4, IL-6, IL-10, and IL-12p70, in venous blood and BALF of a total of 33 patients with lung cancer and 33 patients with benign lung diseases. Significant differences were found between the two groups in various clinical parameters. The cytokine levels were significantly higher among patients with malignant disease, while the BALF analysis revealed higher cytokine levels compared with serum analysis. It was discovered that the levels of cancer-specific cytokines in the lavage fluid increased significantly sooner and were present at a greater concentration than those in the peripheral blood. After one month of treatment, the serum markers decreased significantly but slower in the lavage fluid. The differences between serum and BALF markers remained significant. It was observed that the highest correlation was among IL-6 (serum) and IL-6 (lavage), with a coefficient of 0.774 (*p*-value < 0.001), and IL-1 (serum) and IL-1β (lavage), with a coefficient value of 0.610 (*p*-value < 0.001). Other significant correlations among serum and lavage cytokines were observed between IL-6 (lavage) and IL-1 (serum) (rho = 0.631, *p*-value < 0.001) and CRP (rho = 0.428, *p*-value = 0.001), respectively. This study revealed significant differences and correlations in clinical parameters, serum markers, and BALF inflammatory markers between patients with lung cancer and those with benign lung pathologies. The results highlight the importance of understanding the inflammatory profiles of these conditions and could contribute to the development of targeted therapies or diagnostic approaches in the future. Further research is needed to validate these findings, explore their implications for clinical practice, and determine the diagnostic and prognostic value of these cytokines for lung cancer.

## 1. Introduction

The number of people diagnosed with lung cancer and those who pass away from the condition is rising worldwide. It is the leading cause of death from cancer in both men and women [1], and it is the most prevalent cause of cancer overall [2,3]. Considering the many therapy techniques that are now available, the 5-year survival rate for lung cancer patients is about 15% [4]. The majority of lung cancer patients are diagnosed at a late stage, which contributes to a poor prognosis for their long-term survival. This is because there are not enough clinical tests that are noninvasive that can be used for early diagnosis and screening of these individuals [5,6]. Due to this, it is essential to locate certain biomarkers in order to arrive at an accurate diagnosis as quickly as feasible [5].

According to the findings of a number of studies, chronic inflammation performs a significant part in the process of carcinogenesis. This is true not only in the beginning stages of the malignant process but also in later phases, such as malignant conversion, invasion, and metastasis [7,8]. The innate immune response and the acquired immune response both serve to illustrate the functional connection that exists between inflammation and cancer. Inflammatory cells and tumor cells both release cytokines and chemokines, which are proteins that have a function in increasing activity in both cells and the humoral system [5,6,9].

There are studies that have shown the utility of detecting certain cytokines from bronchoalveolar lavage fluid or from blood in the differential diagnosis of lung cancer [4,5,6,10]. Inflammatory cytokines, such as IFN- γ, IL-1β, IL-2, IL-4, IL-6, IL-10, IL-12p70, and TNF-α, have been shown to be involved in the immune response associated with cancer progression [11]. IFN-gamma, primarily produced by NK cells and T cells, performs a vital role in enhancing antigen presentation and the cytotoxic activity of T cells. IL-1β, a pro-inflammatory cytokine, is known to promote angiogenesis and invasiveness of tumor cells. Similarly, IL-2 is a cytokine secreted by T cells in response to antigen stimulation and is crucial for T cell proliferation and NK cell activity. Other interleukins may promote tumor growth by inhibiting effector T-cell function and enhancing regulatory T-cell function [12].

Nevertheless, further research is required to discover the function that cytokines perform in lung cancer and find biomarkers that may be used for the diagnosis, evaluation of prognosis, and assessment of therapy response in patients with this disease. It is hypothesized that TNF-, IFN-, TGF-, VEGF, and interleukins are some of the most important cytokines involved in the pathogenesis of lung cancer and might potentially serve as biomarkers for diagnosis, prognosis, and evaluation of treatment response. Therefore, the purpose of the research was expanded to evaluate the association between the blood levels and BALF levels of inflammatory proteins IFN-γ, TNF-α, IL-1β, IL-2, IL-4, IL-6, IL-10, and IL-12p70, and to compare their levels in patients with lung cancer and patients with benign lung diseases.

## 2. Materials and Methods

### 2.1. Study Design

A prospective study was performed with the purpose of comparing the inflammatory syndrome in patients with bronchopulmonary cancer with a cohort of patients with benign lung pathology. We examined the levels of IFN-γ, TNF-α, IL-1β, IL-2, IL-4, IL-6, IL-10, and IL-12p70 in the blood and from the bronchoalveolar lavage fluid. The purpose of the study and the procedures that needed to be performed were explained to the patients. Informed consent was obtained from all subjects who were willing to participate in the study. The study was conducted according to the guidelines of the Declaration of Helsinki and approved by the Institutional Review Board of the Hospital of Infectious Diseases and Pulmonology “Victor Babes”, from Timisoara, Romania, on 23 September 2022, with the number 10,218.

### 2.2. Study Cohort

Patients admitted to the Pneumology Clinic of the “Victor Babes” Clinical Hospital from Timisoara between September 2022 and February 2023 were included in this study. The inclusion criteria comprised: (1) patients with a major suspicion of bronchopulmonary cancer on the chest computed tomography (CT), (2) the need to perform bronchoscopy for diagnostic purposes, and (3) patients with a Karnofsky performance status ≥ 60% [13].

The inclusion criteria for the controls were: patients with pulmonary imaging changes that required bronchoscopy for diagnostic purposes. The control group included patients with diffuse interstitial pneumopathy (hypersensitivity pneumonitis, sarcoidosis, nonspecific interstitial pneumonia—NSIP), obstructive pulmonary pathologies, and patients with chronic cough. On the other side, the exclusion criteria of all participants were: (1) severe heart failure NYHA III and IV [14], (2) contraindications for bronchoscopy [15,16,17], (3) the patient’s refusal to participate in the study, and (4) patients without endoscopic characteristics of bronchopulmonary cancer [18]. At the end of the study period, 33 patients with suspected bronchopulmonary cancer were included in the study, and 33 patients were included in the control group. All patients and controls underwent bronchoscopy for diagnostic purposes before initiation of treatment. All bronchoscopies were performed by one researcher, based on the hospital guidelines, in the same hospital unit.

The variables considered for inclusion and analysis comprised the following: age, age range, body mass index (BMI), BMI proportions, smoking status, pack-year number, exposure to respiratory hazards, diagnosis of lung pathology in the control group, signs and symptoms (cough, type of cough, thoracic pain, hemoptysis, fever, weight loss, wheezing and stridor, dyspnea, anorexia, and other symptoms), the Modified Medical Research Council (mMRC) scale of dyspnea, pulmonary auscultation, Charlson Comorbidity Index, spirometry measurements, laboratory analysis (CRP, VSH, WBC, neutrophils, IL-6, and ferritin), sputum analysis, BAL analysis (IFN-gamma, IL-1β, IL-2, IL-4, IL-6, IL-10, IL-12p70, and TNF-α). The choice to measure IFN-γ, TNF-α, IL-1β, IL-2, IL-4, IL-6, IL-10, and IL-12p70 was primarily influenced by the practical considerations associated with the available resources for this study, and the rationale of cost-effectiveness and relevance.

### 2.3. Interventions and Definitions

Bronchoscopies were performed by the same bronchoscopist according to a standardized technique. During the bronchoscopy, the patients were awake, and local anesthesia (topical) with 10% lidocaine was performed. Patients were monitored during bronchoscopy by pulse oximetry. In patients with suspected bronchopulmonary neoplasm, the bronchoscope was inserted and fixed at the segmental or subsegmental level of the affected lobe (where the tumor formation was present), the lavage being performed before the collection of the biopsy fragments. In patients from the control group, the lavage was performed at the level of the right middle lobe or the lingula in the case of a diffuse lesion aspect or at the level of the lobe where the CT lesions predominated.

The lavage was performed with 120 mL of sterile saline solution (NaCl 0.9%), which was heated to room temperature. The instillation was carried out fractionally in aliquots of 20 mL. After each installation, the liquid was immediately recovered by gentle suction in a wedged position. The maneuver was repeated six times, recovering approximately 60–80% of the instilled liquid. The liquid from the first aliquot was not mixed with the others, being sent for bacteriological examination.

The recovered liquid was filtered through cheesecloth. A total of 2 mL of liquid was taken, which was centrifuged for 15 min at 1000 G, after which they were stored at −70 °C until cytokine analysis was performed. The remaining liquid was centrifuged for 15 min at 2000 G, after which the sediment was recovered in 2 mL of transport medium. Once recovered, it was centrifuged for 3 min at 3000 G, and a cytological examination was performed from the sediment by staining with May-Grünwald Giemsa (MGG). The biopsy fragments collected from patients with lung tumors were fixed in formalin and sent for histopathological and immunohistochemical examination.

Patients’ treatment in the context of this study refers to the therapeutic interventions that were initiated for the patients based on their individual diagnoses following bronchoscopy and subsequent investigations. For patients with lung cancer, treatment after bronchoscopy and diagnosis involves surgery, radiation, chemotherapy, immunotherapy, or a combination of these, as per the standard of care for their specific stage and type of lung cancer. The benign lung disease patients underwent appropriate management for their specific conditions, such as medication for inflammation or infection or other necessary interventions. The measurements of cytokine levels were repeated one month after the initiation of these treatments. This was done to investigate the potential impact of these therapeutic interventions on the inflammatory profiles of patients. By comparing the cytokine levels before and after treatment, we aimed to understand whether these interventions could modulate the inflammatory response, as evidenced by changes in the cytokine levels.

### 2.4. Laboratory Analysis

Laboratory evaluation was performed by obtaining peripheral venous blood samples on the day of bronchoscopy, which were then collected using sterile tubes and promptly transported to the laboratory for processing. Cytokines in the lavage fluid were analyzed using the enzyme-linked immunosorbent assay (ELISA) method, which utilizes the fully cartridge-based system ELLA. This device enables simultaneous measurement of multiple analytes in various sample types. We utilized commercially available sandwich ELISA kits for analysis. Prior to analysis, all collected samples were thawed and centrifuged for 15 min at 1000× *g*, and the supernatant was subsequently diluted four times by adding 20 µL to 60 µL of sample diluent. Interleukins from venous blood were determined using the electrochemiluminescence (ECLIA) method.

### 2.5. Statistical Analysis

GraphPad Prism for Microsoft Windows, version 6.0, was used to conduct the statistical analysis (GraphPad Software, Boston, MA, USA). The Kolmogorov–Smirnov test was used to assess the normality of the data. The mean value, which represents central tendency, and the standard deviation, which measures dispersion, were used to represent normally distributed data. Student’s *t*-test was used to examine the mean difference between the two comparison groups. The median and interquartile range (IQR) were used to characterize non-normally distributed data, presented in box plots, while the Mann–Whitney u-test was used to compare these variables. Considering the frequency assumption for the Chi-square test was not fulfilled, proportions were compared using Fisher’s exact test. A correlation matrix was plotted to observe the association between inflammatory markers, and their statistical significance was represented by the correlation coefficient “rho” and *p*-value. A *p*-value below 0.05 was regarded as statistically significant.

## 3. Results

### 3.1. Background Analysis

The study included 33 patients with lung cancer and 33 patients with benign lung pathology in the control group. The suspicion of bronchopulmonary cancer was raised by the imaging aspect of the chest CT, and the diagnosis was established by histopathological and immunohistochemical examination of the bronchial biopsy. The mean age of cases was 62.7 ± 8.7 years, while the mean age of controls was 58.2 ± 13.6 years. There was no statistically significant difference in age between the two groups (*p* = 0.114). In terms of body mass index (BMI), the mean BMI of cases was significantly lower than that of controls (23.5 ± 4.1 kg/m^2^ vs. 27.3 ± 5.8 kg/m^2^, *p* = 0.003). Furthermore, the distribution of BMI categories was significantly different between the two groups (*p* = 0.030). Among cases, 54.5% had a BMI between 25 and 29.9 kg/m^2^, and 39.4% had a BMI greater than 30 kg/m^2^. In contrast, 24.2% of controls had a BMI between 25 and 29.9 kg/m^2^, and 57.6% had a BMI greater than 30 kg/m^2^.

Gender distribution showed a higher percentage of males in the cases group (63.6%) compared to the controls group (42.4%), but the difference was not statistically significant (*p* = 0.084). Regarding smoking history, 60.6% of cases were either current or ex-smokers, compared to 48.5% of controls. However, this difference was not statistically significant (*p* = 0.322). The median pack-year smoking history was significantly higher in cases (34.0, IQR 22.5–41.0) than in controls (18.5, IQR 12.0–29.5) with a *p*-value of <0.001. The exposure to respiratory hazards was not significantly different between the two groups, with 42.4% of cases and 51.5% of controls reporting exposure (*p* = 0.459). The distribution of benign lung pathologies among controls included asthma (12.1%), chronic bronchitis (27.3%), emphysema (12.1%), interstitial lung disease (24.2%), hypersensitivity pneumonitis (18.2%), and others (6.1%). Among cases, 42.4% had small cell lung cancer (SCLC), and 57.6% had non-small cell lung cancer (NSCLC), as presented in Table 1.

### 3.2. Clinical Analysis

Table 2 presents the clinical data of study participants, including 33 cases of lung cancer and 33 controls with benign lung pathology. The majority of cases (87.9%) and controls (78.8%) reported cough, with no significant difference between the two groups (*p* = 0.321). The type of cough was predominantly dry in both cases (69.0%) and controls (65.4%), and this difference was not statistically significant (*p* = 0.777). Thoracic pain was reported in 30.3% of cases and 6.1% of controls, with a statistically significant difference between the two groups (*p* = 0.010). Hemoptysis was reported in 18.2% of cases and 9.1% of controls, but this difference was not statistically significant (*p* = 0.281). Fever was reported in 3.0% of cases and 9.1% of controls, with no significant difference (*p* = 0.302). Notably, weight loss was significantly more frequent in cases (69.7%) than in controls (6.1%, *p* < 0.001).

Dyspnea was reported in 84.8% of cases and 75.8% of controls, with no significant difference (*p* = 0.353). The distribution of mMRC dyspnea scores (3–4) was also not significantly different between cases (32.1%) and controls (40.0%, *p* = 0.551). Anorexia was not reported in cases but was present in 54.5% of controls, with a highly significant difference between the groups (*p* < 0.001). Fatigue was reported in 90.9% of cases and 78.8% of controls, with no significant difference (*p* = 0.969). Wheezing and stridor were significantly more frequent in controls (51.5%) than in cases (15.2%, *p* = 0.002). There was no significant difference in the percentage of normal pulmonary auscultation findings between cases (54.5%) and controls (33.3%, *p* = 0.083). The mean duration of symptom onset was significantly shorter in cases (5.6 ± 3.7 months) compared to controls (15.2 ± 10.4 months, *p* < 0.001). A Charlson Comorbidity Index (CCI) greater than 2 was significantly more common among cases (63.6%) than controls (36.4%, *p* = 0.026).

Table 3 presents lung function studies in the 33 cases with lung cancer and 33 controls with benign lung pathology. The distribution of spirometry patterns was significantly different between the two groups (*p* < 0.001). Among cases, 15.2% had normal spirometry, 33.3% had an obstructive pattern, 9.1% had a restrictive pattern, and 42.4% had a mixed pattern. In contrast, among controls, 27.3% had normal spirometry, 18.2% had an obstructive pattern, 45.5% had a restrictive pattern, and 9.1% had a mixed pattern. Regarding the degree of respiratory dysfunction based on forced expiratory volume in 1 s (FEV1), there was no significant difference between cases and controls (*p* = 0.173). In some cases, 39.4% had mild dysfunction (FEV1 ≥ 80), 45.5% had moderate dysfunction (FEV1 50–79), and 15.2% had severe dysfunction (FEV1 30–49). Among controls, 54.5% had mild dysfunction, 42.4% had moderate dysfunction, and 3.0% had severe dysfunction.

### 3.3. Laboratory Analysis

Table 4 presents laboratory analysis of serum markers in the 33 cases with lung cancer and 33 controls with benign lung pathology, both at the initial evaluation and one month after treatment. At the initial evaluation, there were statistically significant differences between cases and controls for all serum markers. The mean levels of C-reactive protein (CRP) were markedly higher in cases (76.6 ± 54.4 mg/L) compared to controls (8.5 ± 6.8 mg/L, *p* < 0.001). Similarly, the mean erythrocyte sedimentation rate (ESR) was significantly higher in cases (63.8 ± 34.3 mm/h) than in controls (22.5 ± 14.1 mm/h, *p* < 0.001). Cases also exhibited significantly higher mean levels of leucocytes (11.4 ± 4.8 × 10^3^ vs. 7.5 ± 2.3 × 10^3^, *p* < 0.001), neutrophils (74.0 ± 8.8% vs. 60.9 ± 8.3%, *p* < 0.001), interleukin-1 (IL-1; 29.3 ± 18.4 pg/mL vs. 7.2 ± 4.8 pg/mL, *p* < 0.001), interleukin-6 (IL-6; 31.6 ± 20.9 pg/mL vs. 5.8 ± 3.9 pg/mL, *p* < 0.001), and ferritin (504.7 ± 265.1 ug/L vs. 215.4 ± 132.1 ug/L, *p* < 0.001).

**Figure 1:**
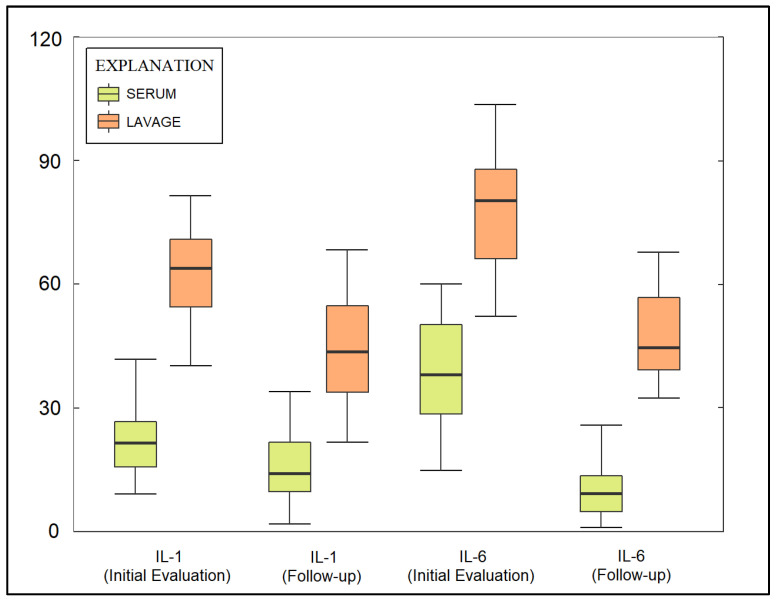
Boxplot of serum and lavage fluid evaluation of IL-1 and IL-6 at initial evaluation and follow-up.

One month after treatment, all serum markers remained significantly different between cases and controls. The mean CRP levels were still higher in cases (51.4 ± 35.9 mg/L) compared to controls (6.6 ± 4.1 mg/L, *p* < 0.001). The mean ESR remained significantly higher in cases (42.8 ± 25.2 mm/h) compared to controls (14.9 ± 8.3 mm/h, *p* < 0.001). Similarly, cases exhibited higher mean levels of leucocytes (8.8 ± 3.6 × 10^3^ vs. 6.4 ± 2.2 × 10^3^, *p* = 0.002), neutrophils (63.5 ± 7.1% vs. 59.3 ± 8.4%, *p* < 0.001), IL-1 (15.4 ± 13.8 pg/mL vs. 6.3 ± 3.3 pg/mL, *p* < 0.001), IL-6 (12.9 ± 11.6 pg/mL vs. 5.7 ± 3.5 pg/mL, *p* < 0.001), and ferritin (421 ± 247.3 ug/L vs. 226.3 ± 142.8 ug/L, *p* < 0.001), as seen in Figure 1.

Table 5 presents the bronchial lavage analysis of inflammatory markers in the 33 cases with lung cancer and 33 controls with benign lung pathology, both at the initial evaluation and one month after treatment. At the initial evaluation, there were statistically significant differences between cases and controls for most inflammatory markers. The mean interferon-gamma (IFN-γ) levels were significantly lower in cases (69.7 ± 30.6 pg/mL) compared to controls (124.3 ± 58.1 pg/mL, *p* < 0.001). Similarly, the mean interleukin-1b (IL-1β) levels were significantly higher in cases (98.8 ± 33.1 pg/mL) than in controls (24.7 ± 11.5 pg/mL, *p* < 0.001). The mean levels of IL-4 (53.5 ± 20.1 pg/mL vs. 38.0 ± 15.2 pg/mL, *p* = 0.008), IL-6 (126.2 ± 61.8 pg/mL vs. 44.3 ± 28.9 pg/mL, *p* < 0.001), IL-10 (42.7 ± 22.5 pg/mL vs. 25.8 ± 13.9 pg/mL, *p* = 0.005), IL-12p70 (106.2 ± 67.3 pg/mL vs. 72.0 ± 42.1 pg/mL, *p* = 0.016), and tumor necrosis factor-alpha (TNF-α; 45.6 ± 15.9 pg/mL vs. 85.3 ± 33.7 pg/mL, *p* < 0.001) also exhibited significant differences between cases and controls. The difference in mean IL-2 levels was not statistically significant (32.4 ± 15.7 pg/mL vs. 26.1 ± 9.6 pg/mL, *p* = 0.053).

It was discovered that the levels of cancer-specific cytokines in the lavage fluid increased significantly sooner and were present at a greater concentration than those in the peripheral blood. One month after treatment, significant differences between cases and controls persisted for most inflammatory markers, except for IFN-γ (57.2 ± 26.8 pg/mL vs. 68.8 ± 32.9 pg/mL, *p* = 0.121). The mean levels of IL-1β (69.6 ± 29.3 pg/mL vs. 15.2 ± 9.7 pg/mL, *p* < 0.001), IL-2 (28.2 ± 13.7 pg/mL vs. 13.9 ± 8.5 pg/mL, *p* < 0.001), IL-4 (44.0 ± 21.6 pg/mL vs. 28.4 ± 18.2 pg/mL, *p* = 0.002), IL-6 (97.3 ± 50.9 pg/mL vs. 39.2 ± 30.8 pg/mL, *p* < 0.001), IL-10 (38.5 ± 19.3 pg/mL vs. 19.8 ± 11.7 pg/mL, *p* < 0.001), IL-12p70 (81.1 ± 42.0 pg/mL vs. 52.3 ± 29.1 pg/mL, *p* < 0.001), and TNF-α(34.9 ± 16.5 pg/mL vs. 47.1 ± 22.4 pg/mL, *p* = 0.014) all demonstrated significant differences between cases and controls.

**Figure 2:**
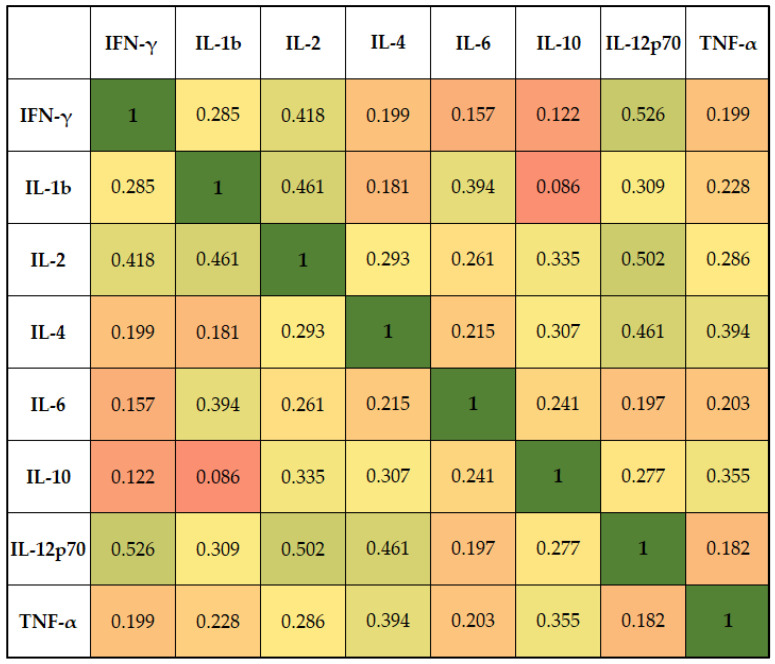
Correlation matrix of cytokines from bronchoalveolar lavage fluid.

The correlation analysis presented in Figure 2 identified multiple significant correlations between cytokines from bronchoalveolar lavage fluid. Interleukin-2 levels correlated significantly with IFN-γ (rho = 0.418), IL-1b (rho = 0.461), IL-10 (rho = 0.335), and IL-12p70 (rho = 0.502) (*p*-value < 0.05). Interleukin-4 was positively correlated with IL-10 (rho = 0.335) and IL-12p70 (rho = 0.502), while Interleukin-6 was significantly correlated only with IL-1b (rho = 0.461, *p*-value < 0.001). Other significant positive correlations were identified between IL-10, IL-4 (rho = 0.307), and TNF-alpha (rho = 0.355), respectively, and IL-12p70 and IFN-γ (rho = 0.526).

Regarding associations between cytokines in the lavage fluid and serum levels, it was observed that the highest correlation was among IL-6 (serum) and IL-6 (lavage), with a coefficient of 0.774 (*p*-value < 0.001), and IL-1 (serum) and IL-1β (lavage), with a “rho” value of 0.610 (*p*-value < 0.001). Other significant correlations among serum and lavage cytokines were observed between IL-6 (lavage) and IL-1 (serum) (rho = 0.631, *p*-value < 0.001) and CRP (rho = 0.428, *p*-value = 0.001), respectively.

## 4. Discussion

### 4.1. Literature Findings

Lung cancer is the main cause of morbidity and mortality among malignant diseases. The poor survival of these patients is largely due to the late diagnosis [19,20]. The screening methods used, such as chest X-ray and sputum cytological examination, do not increase the survival of patients with lung cancer. Annual low-dose CT screening in high-risk individuals has shown a decrease in lung cancer mortality, but additional studies are needed to establish the benefit of this type of screening and the time intervals at which it should be performed [21,22]. The identification and development of biomarkers for the early diagnosis of lung cancer could increase the survival of these patients [6].

Studies suggest that chronic inflammation favors the development and progression of lung cancer. Rudolf Virchow was the one who proposed for the first time the role of chronic inflammation in the development of cancer in 1863. Virchow’s hypothesis was based on the identification of inflammatory cells in resected tumors, and on the observation that neoplastic cells developed more frequently in sites with chronic inflammation [8,23,24,25]. The development of neoplastic cells in the presence of chronic inflammation requires the existence of cytokines, chemokines, reactive oxygen species, and the activation of important transcription factors. Inflammatory cells, and tumor cells, secrete cytokines, which is why circulating cytokines could be biomarkers used for the early detection of lung cancer [20,24,25]. Increased cytokine levels in BALF or serum of lung cancer patients have been observed in several studies. Among the cytokines studied are TNF-α, IFN-γ, TGF-β, VEGF, IL-6, IL-8, IL-10, and IL-1β [4,5,6,19,26].

IL-6 is an important, multifunctional proinflammatory cytokine that has a role in regulating the immune response, inflammation, hematopoiesis, and oncogenesis. Dysregulation of the production of this cytokine is involved in the pathogenesis of several diseases, including lung cancer. IL-6 and IL-6 receptors have roles in the growth and differentiation of tumor cells and in angiogenesis through the JAK (Janus kinase)-STAT signaling pathway [5,27,28,29]. IL-1β is another cytokine overexpressed in lung tumors and is involved in tumor development and metastasis by inducing growth factors, such as vascular endothelial growth factor, prostaglandin E2 (PGE2), and transforming growth factor β [30,31].

Identifying biomarkers to diagnose lung cancer and differentiate between benign and malignant lung diseases is important for patient management. In the present study, we conducted a prospective study to investigate whether levels of BALF IL-6 and IL-1β and levels of serum IL-6 could be useful in distinguishing malignant from benign pulmonary diseases. The levels of BALF IL-6 and IL-1β were determined by commercially available sandwich ELISA kits; the blood IL-6 was determined by electrochemiluminescence. The results showed that the levels of IL-6 in the blood were higher among patients with lung cancer than in patients with benign diseases. We did not find any correlation between BALF IL-6 and blood IL-6 in the studied groups. Additionally, no differences in BALF for IL-1b were observed between lung cancer patients and non-cancer controls.

In a study led by Pine SR., it was shown that IL-6 in the serum had high levels in patients diagnosed with lung cancer. Additionally, the values of this cytokine were increased in patients who subsequently developed lung cancer up to 2 years before the onset of the disease [19]. Increased values of IL-6 in the serum of cancer patients have also been identified in other studies [4,32,33]. Several studies have shown that patients with lung cancer and elevated values of IL-6 in the serum have a worse prognosis [34,35,36].

There are studies that have shown the usefulness of dosing certain cytokines in BALF for the differential diagnosis of lung cancer [4,37,38]. In contrast to the results obtained in our research, other studies obtained significantly higher values of IL-6 and IL-1β in BALF in patients with lung cancer compared to the control group [4]. Chen Z. et al., in their study, did not obtain different values of IL-6 in BALF between patients with lung cancer and those with benign lung diseases, their results being consistent with what we obtained in our study [6]. In a study in which the levels of IL-6 and IL-1βin the serum were measured, it was shown that patients with lung cancer had higher levels of IL-6, regardless of race. Additionally, the study showed that IL-1β values in the serum were significantly higher in patients with lung cancer but of African American race [20].

In a past study, researchers conducted a prospective investigation to determine whether levels of bronchoalveolar lavage (BAL) fluid TGF-β1, IL-6, and TNF-α could differentiate between malignant and benign pulmonary diseases [6]. Patients with suspected lung cancer were enrolled, and the levels of these markers were determined using commercially available sandwich ELISA kits. The results demonstrated that TGF-β1 levels were higher in patients with lung cancer compared to those with benign diseases, with a significant correlation found between TGF-β1 and IL-6 in BALF. However, no significant differences in BAL IL-6 or TNF-α were observed between the two groups.

Further analysis showed that TGF-β1 expression was significantly higher in lung cancer patients, suggesting that it could serve as a useful biomarker for diagnosing the disease. ROC analysis was conducted to examine the diagnostic ability of TGF-β1 for predicting lung cancer, and the results revealed a diagnostic threshold of 10.85 pg/mL [6]. With this threshold, TGF-β1 exhibited a sensitivity of 62.2%, a specificity of 60.6%, a positive predictive value of 67.5%, and a negative predictive value of 52.6% in predicting malignancy. Therefore, low levels of TGF-β1 in BALF indicated a low probability of malignancy. Despite existing evidence supporting the functional link between TGF-β1 and IL-6 in various human diseases, the study found no elevated concentrations of IL-6 in BALF for lung cancer patients, although a significant correlation between TGF-β1 and IL-6 in BALF was observed [39,40].

Lastly, the study investigated the potential of TNF-α as a diagnostic marker for lung cancer. While previous research found elevated levels of TNF-α in both serum and exhaled breath condensate (EBC) of lung cancer patients, the study under discussion did not find any significant differences in TNF-α levels in BALF between benign and malignant groups [41,42]. Furthermore, no significant correlation between TNF-α and TGF-β1 in BALF was discovered. The results ultimately suggested that determining BAL fluid TNF-α levels in flexible bronchoscopy may be unhelpful in diagnosing lung cancer. Moreover, no significant difference in cytokine levels was observed between SCLC and NSCLC patients, although studies with larger cohorts can consider this for further analysis.

In the context of using cytokines as potential biomarkers for lung cancer, it is important to consider the significant inter-individual variability observed in our study. This variability could potentially limit the application of cytokines as standalone markers and necessitates careful interpretation when applied to individual patients. Larger studies are needed to establish reference ranges or cutoff values for BAL and serum cytokines that differentiate benign from malignant lung pathologies. Furthermore, the intra-individual variability of these markers should be explored by obtaining repeated measures from the same patient over time. It is also important to consider the possibility of integrating cytokine levels with other clinical, radiological, and molecular data to improve diagnostic accuracy.

In the current study, there were significant differences observed in the levels of multiple interleukins and other cytokines between lung cancer patients and those with benign lung pathology. Such results suggest that these cytokines could potentially serve as biomarkers not only for diagnosing lung cancer but also for distinguishing malignant from benign lung pathologies. Thus, they might aid in avoiding unnecessary invasive procedures in patients with benign conditions, thereby improving patient care. Moreover, cytokines’ concentrations may also have prognostic value, such as the high levels of IL-6 that were previously associated with a worse prognosis in multiple studies. Therefore, these cytokines might also serve as indicators of disease progression and overall survival, aiding in clinical decision-making and patient management.

Additionally, our findings primarily provide an overview of cytokine present in the BALF and serum without distinguishing their cellular origin. Thus, the exploration of cytokine production at a cellular level would offer a more comprehensive understanding of the dynamics within the tumor microenvironment. This type of investigation would require more specific techniques, such as cell sorting followed by single-cell RNA sequencing or intracellular cytokine staining followed by flow cytometry [43], which were beyond the scope of this study.

### 4.2. Study Strengths and Limitations

Our study has some limitations. First, the study included 33 patients with suspected bronchopulmonary cancer and 33 patients in the control group. The relatively small sample size may limit the generalizability of the results and reduce the study’s statistical power. The study design did not involve randomization or blinding, which could lead to selection bias or influence the interpretation of the results by the researchers. The inclusion and exclusion criteria might have led to selection bias, as patients with severe heart failure, contraindications for bronchoscopy, or without endoscopic characteristics of bronchopulmonary cancer were excluded from the study. Additionally, several factors, such as age, BMI, smoking status, and exposure to respiratory hazards, were considered for inclusion and analysis. Lastly, the study used different methods to measure cytokines in the blood and lavage fluid, which may introduce variability and affect the comparability of the results. The blood samples were analyzed using the electrochemiluminescence (ECLIA) method, while the lavage fluid samples were analyzed using the enzyme-linked immunosorbent assay (ELISA) method. Other potentially important cytokines, such as TGF-b1, that were shown to have an important correlation with IL-6 in lung cancer diagnosis were not included in this study due to limited funds and cost-effectiveness considerations.

The novelty of our research lies primarily in its comprehensive approach, evaluating both BALF and serum levels of multiple cytokines (of IFN-γ, TNF-α, IL-1β, IL-2, IL-4, IL-6, IL-10, and IL-12p70), providing a more holistic understanding of the inflammatory response associated with lung pathologies. While previous studies have focused on a select few cytokines, our study broadens the scope, adding value to the existing body of research in lung cancer diagnostics. Furthermore, our comparative analysis between patients with malignant and benign lung diseases helps identify potential biomarkers for differential diagnosis, thereby addressing a persistent challenge in clinical practice. Another unique aspect of our work is its emphasis on the significant inter-individual variability in cytokine expression. Overall, these findings underscore the importance of personalized diagnostic and treatment strategies in managing lung diseases.

## 5. Conclusions

In conclusion, this study demonstrates notable differences and correlations in clinical parameters, serum markers, and bronchoalveolar lavage fluid inflammatory markers between patients with lung cancer and those with benign lung diseases. The findings underscore the significance of understanding the distinct inflammatory profiles of these conditions, as they may potentially inform the development of targeted therapies or diagnostic approaches. Specifically, the study revealed that cancer-specific cytokines in the lavage fluid increased significantly earlier and were present at higher concentrations than those in peripheral blood. The highest correlations were found between IL-6 in serum and IL-6 in the lavage fluid, and IL-1 in serum and BALF IL-1β. These results could have implications for clinical practice, including diagnostic and prognostic value for lung cancer. However, further research is necessary to validate these findings and explore their clinical implications in greater depth, and to determine the diagnostic and prognostic value of these cytokines for lung cancer.

## Figures and Tables

**Table 1:** Background data of study participants.

Variables	Cases (*n* = 33)	Controls (*n* = 33)	*p*-Value
Age (mean ± SD)	62.7 ± 8.7	58.2 ± 13.6	0.114
Age range	48–75	37–73	-
BMI (mean ± SD)	23.5 ± 4.1	27.3 ± 5.8	0.003
BMI categories			0.030
18.5–24.9 (kg/m^2^)	2 (6.1%)	6 (18.2%)	
25–29.9 (kg/m^2^)	18 (54.5%)	8 (24.2%)	
>30 (kg/m^2^)	13 (39.4%)	19 (57.6%)	
Gender (male, %)	21 (63.6%)	14 (42.4%)	0.084
Smoker/Ex-smoker (yes, %)	20 (60.6%)	16 (48.5%)	0.322
Pack-year smoking (median, IQR)	34.0 (22.5–41.0)	18.5 (12.0–29.5)	<0.001
Exposure to respiratory hazards (yes, %)	14 (42.4%)	17 (51.5%)	0.459
Benign lung pathology			
Asthma	-	4 (12.1%)	-
Chronic bronchitis	-	9 (27.3%)	-
Emphysema	-	4 (12.1%)	-
ILD	-	8 (24.2%)	-
HP	-	6 (18.2%)	-
Others	-	2 (6.1%)	-
Malignant lung pathology			
SCLC	14 (42.4%)	-	-
NSCLC	19 (57.6%)	-	-

BMI—Body Mass Index; SD—Standard Deviation; IQR—Interquartile Range; ILD—Interstitial Lung Disease; HP—Hypersensitivity Pneumonitis; SCLC—Small Cell Lung Cancer; NSCLC—Non-Small Cell Lung Cancer.

**Table 2:** Clinical data of study participants.

Signs and Symptoms	Cases (*n* = 33)	Controls (*n* = 33)	*p*-Value
Cough (*n*, %)	29 (87.9%)	26 (78.8%)	0.321
Type of cough (dry, %)	20 (69.0%)	17 (65.4%)	0.777
Thoracic pain (*n*, %)	10 (30.3%)	2 (6.1%)	0.010
Hemoptysis (*n*, %)	6 (18.2%)	3 (9.1%)	0.281
Fever (*n*, %)	1 (3.0%)	3 (9.1%)	0.302
Weight loss (*n*, %)	23 (69.7%)	2 (6.1%)	<0.001
Dyspnea (*n*, %)	28 (84.8%)	25 (75.8%)	0.353
mMRC dyspnea (3–4)	9 (32.1%)	10 (40.0%)	0.551
Anorexia (*n*, %)	0 (0.0%)	18 (54.5%)	<0.001
Fatigue (*n*, %)	30 (90.9%)	26 (78.8%)	0.969
Wheezing and stridor (*n*, %)	5 (15.2%)	17 (51.5%)	0.002
Pulmonary auscultation (normal, %)	18 (54.5%)	11 (33.3%)	0.083
Symptom onset, months (mean ± SD)	5.6±3.7	15.2 ± 10.4	<0.001
Lung cancer staging			
IB	6 (18.2%)	–	
IIA	14 (42.4%)	–	
IIB	9 (27.3%)	–	
IIIA	4 (12.1%)	–	
CCI > 2	21 (63.6%)	12 (36.4%)	0.026

COPD—Chronic Obstructive Pulmonary Disease; SD—Standard Deviation; mMRC—modified Medical Research Council; CCI—Charlson Comorbidity Index.

**Table 3:** Lung function studies.

Variables	Cases (*n* = 33)	Controls (*n* = 33)	*p*-Value
Spirometry			<0.001
Normal	5 (15.2%)	9 (27.3%)	
Obstructive pattern	11 (33.3%)	6 (18.2%)	
Restrictive pattern	3 (9.1%)	15 (45.5%)	
Mixt pattern	14 (42.4%)	3 (9.1%)	
Degree of respiratory dysfunction (FEV1)			0.173
Mild (≥80)	13 (39.4%)	18 (54.5%)	
Moderate (50–79)	15 (45.5%)	14 (42.4%)	
Severe (30–49)	5 (15.2%)	1 (3.0%)	

SCC—Squamous Cell Cancer; ACC—Adenocarcinoma; SCLC—Small Cell Lung Cancer; PD-L1—Programmed cell Death Ligand 1; ALK—Anaplastic Lymphoma Receptor Tyrosine Kinase Gene; EGFR—Epidermal Growth Factor Receptor; FEV—Forced Expiratory Volume.

**Table 4:** Laboratory analysis.

Serum Markers	Normal Range	Cases (*n* = 33)	Controls (*n* = 33)	*p*-Value
Initial evaluation				
CRP	0–5 mg/L	76.6 ± 54.4	8.5 ± 6.8	<0.001
ESR	3–10 mm/h	63.8 ± 34.3	22.5 ± 14.1	<0.001
Leucocytes	4–10 × 10^3^	11.4 ± 4.8	7.5 ± 2.3	<0.001
Neutrophils	55–65%	74.0 ± 8.8	60.9 ± 8.3	<0.001
IL-1	0–5 pg/mL	29.3 ± 18.4	7.2 ± 4.8	<0.001
IL-6	0–7 pg/mL	31.6 ± 20.9	5.8 ± 3.9	<0.001
Ferritin	30–400 ug/L	504.7 ± 265.1	215.4 ± 132.1	<0.001
1 month after treatment				
CRP	0–5 mg/L	51.4 ± 35.9	6.6 ± 4.1	<0.001
ESR	3–10 mm/h	42.8 ± 25.2	14.9 ± 8.3	<0.001
Leucocytes	4–10 × 10^3^	8.8 ± 3.6	6.4 ± 2.2	0.002
Neutrophils	55–65%	63.5 ± 7.1	59.3 ± 8.4	<0.001
IL-1	0–5 pg/mL	15.4 ± 13.8	6.3 ± 3.3	<0.001
IL-6	0–7 pg/mL	12.9 ± 11.6	5.7 ± 3.5	<0.001
Ferritin	30–400 ug/L	421 ± 247.3	226.3 ± 142.8	<0.001

CRP—C-reactive Protein; ESR—Erythrocyte Sedimentation Rate; IL—Interleukin.

**Table 5:** Bronchial lavage analysis.

Inflammatory Markers	Normal Range *	Cases (*n* = 33)	Controls (*n* = 33)	*p*-Value
Initial evaluation				
IFN-γ (3rd gen.)	<2 pg/mL	69.7 ± 30.6	124.3 ± 58.1	<0.001
IL-1β	<12 pg/mL	98.8 ± 33.1	24.7 ± 11.5	<0.001
IL-2	<5 pg/mL	32.4 ± 15.7	26.1 ± 9.6	0.053
IL-4	<5 pg/mL	53.5 ± 20.1	38.0 ± 15.2	0.008
IL-6 (2nd gen.)	5–15 pg/mL	126.2 ± 61.8	44.3 ± 28.9	<0.001
IL-10	<5 pg/mL	42.7 ± 22.5	25.8 ± 13.9	0.005
IL-12p70	<3 pg/mL	106.2 ± 67.3	72.0 ± 42.1	0.016
TNF-α	<16 pg/mL	45.6 ± 15.9	85.3 ± 33.7	<0.001
1 month after treatment				
IFN-γ (3rd gen.)	<2 pg/mL	57.2 ± 26.8	68.8 ± 32.9	0.121
IL-1β	<12 pg/mL	69.6 ± 29.3	15.2 ± 9.7	<0.001
IL-2	<5 pg/mL	28.2 ± 13.7	13.9 ± 8.5	<0.001
IL-4	<5 pg/mL	44.0 ± 21.6	28.4 ± 18.2	0.002
IL-6 (2nd gen.)	5–15 pg/mL	97.3 ± 50.9	39.2 ± 30.8	<0.001
IL-10	<5 pg/mL	38.5 ± 19.3	19.8 ± 11.7	<0.001
IL-12p70	<3 pg/mL	81.1 ± 42.0	52.3 ± 29.1	<0.001
TNF-α	<16 pg/mL	34.9 ± 16.5	47.1 ± 22.4	0.014

* Compared with normal serum levels (data presented as *n* (%) of samples outside the normal range); IFN—Interferon; IL—Interleukin; TNF—Tumor Necrosis Factor; PCR—Polymerase Chain Reaction.

## Data Availability

Data available on request.
